# Effect of Compound Planting Mode on Nutrient Distribution in Cotton

**DOI:** 10.3390/plants14071051

**Published:** 2025-03-28

**Authors:** Lirong He, Lei Shi, Qiaoni Gao, Guobin Liu, Chutao Liang

**Affiliations:** 1 The Research Center of Soil and Water Conservation and Ecological Environment, Chinese Academy of Sciences and Ministry of Education, Yangling 712100, China; helirong22@mails.ucas.ac.cn; 2 Institute of Soil and Water Conservation, Chinese Academy of Sciences and Ministry of Water Resources, Yangling 712100, China; 3 University of Chinese Academy of Sciences, Beijing 100049, China; 4 Institute of Land Engineering and Technology, Shaanxi Provincial Land Engineering Construction Group Co., Ltd., Xi’an 710075, China; shilei1019@stu.xjtu.edu.cn; 5 Shangluo Drug Inspection Institute, Shangluo 726000, China; ysx_gqn@163.com; 6 College of Environmental Science and Engineering, Shanxi Institute of Science and Technology, Jincheng 048000, China

**Keywords:** composite planting pattern, allometry distribution, stoichiometric ratio, nutrient-use efficiency

## Abstract

Composite planting has become one of the primary agricultural practices promoted in recent years, especially in the northwest inland cotton regions of China, where various economic trees and crops are intercropped with cotton. However, research on the microclimatic differences affecting cotton growth and the nutrient allocation strategies for cotton’s key economic organs (i.e., seed, batt, and shell) in strip composite cropping systems remains limited. In this study, we examined the nutrient allocation strategies of cotton under multiple composite cropping patterns and proposed the most suitable cultivation patterns for this region in the northwest inland region of China, utilizing an allometry partitioning index and ecological stoichiometry, based on a long-term positional experiment. The results revealed that the nutrient distribution of cotton was of equal speed with the combined planting with trees, while there was an allometric distribution index of N and P between the combined planting with maize. The effect of the compound planting mode on the nutrient-use efficiency of cotton was mainly reflected in the organ differentiation stage of its reproductive growth stage. Specifically, cotton showed lower nutrient-use efficiency in reproductive organs when intercropped with low shrubs and herbaceous crops, likely due to the insufficient protective capacity of these plants for cotton. Interestingly, strip intercropping with tall trees improved cotton’s nutrient-utilization efficiency. However, it also resulted in reduced nitrogen and phosphorus content in cotton batt. Moreover, soil indicators such as available nitrogen and electrical conductivity positively influenced the nutrient uptake of cotton shells and roots, while soil phosphorus promoted the nutrient absorption of cotton seed but inhibited the nitrogen and phosphorus of cotton shell and the nitrogen of cotton batt. These findings suggest that nutrient partitioning in cotton is influenced by a variety of soil factors. According to these results, the combined planting pattern of cotton and apple trees should be considered in practice to improve cotton yield and economic benefits in the northwest inland region of China.

## 1. Introduction

Compound planting has emerged as one of the primary agricultural practices promoted in recent years [1,2]. It is widely believed that compound planting can reduce the spread of pests and diseases, while simultaneously enriching crop diversity and enhancing risk resilience [3]. For example, intercropping corn and soybean not only offers clear yield advantages but also improves land-use efficiency and mitigates soybean lodging [4,5,6]. As a legume, soybean possesses nitrogen-fixing properties, making it a valuable fertilizer supplement [7]. However, studies have shown that intercropping with eucalyptus may negatively affect the yield of understory crops [8].

In the northwest inland cotton region of China, the key factors influencing cotton production are the variety and environmental conditions [9,10]. To increase cotton production while integrating widely cultivated cash crops such as Batangas, cotton in this region is often intercropped with tree species such as peach, apple, and Batangas, or with grain crops like maize [4,11]. These practices do not provide fertilizer supplementation but may affect the radiative transfer from farmland [12]. Furthermore, research has indicated that compound planting with cotton can reduce the water-use efficiency of jujube and decrease the photosynthetic rate of cotton [13,14,15]. In the Tarim Basin, intercropping cotton with apricot trees has been shown to influence cotton quality, with apricot tree spacing being a contributing factor [16]. On a positive note, strip intercropping of cotton with wheat has been found to reduce the incidence of cotton bollworm [17,18]. Past studies on environmental influences on cotton growth have primarily focused on climatic differences, soil variations due to fertilizers and irrigation, with a specific emphasis on apricot–cotton intercropping [16]. However, in the inland cotton region of Northwest China, a diverse range of economic tree species and crops are intercropped with cotton, and there has been limited research on the microclimatic effects on cotton growth within strip composite cropping systems [19].

Heterozygous allocation, also known as heterozygous growth, is a common phenomenon in plants [20]. It refers to the differential growth rates of different plant organs or the varying rates of nutrient accumulation across plant organs [21]. The rate of heterozygous allocation is influenced by the plant’s growth environment, species, and variety [22]. Biomass allocation strategies reflect the plant’s adaptation to its environment and highlight the environmental effects on plant ecotypes [23,24]. Nutrient partitioning is a key focus of heterozygous growth modeling and has been extensively studied. For example, nitrogen and phosphorus heterogeneity has been observed in plants like moso bamboo and pine, as well as in cotton, where organ-specific nitrogen and phosphorus heterogeneity has been documented [25,26]. Differences in the environment and management practices lead to distinct nitrogen and phosphorus partitioning, and nitrogen addition affects the nitrogen and phosphorus allometry partitioning characteristics in oil pine [27]. However, previous research on cotton nutrient allocation has mainly focused on leaves, stem, and roots, with little attention given to nutrient distribution in cotton after boll opening [28,29]. There has been insufficient investigation into the nutrient allocation strategies of cotton’s primary economic organs—seeds, batt, and shell—while stoichiometric studies on heterozygous growth have yet to be applied to cotton.

To address these gaps, this study examined the nutrient allocation strategies of cotton under multiple composite cropping patterns in Shache County, Xinjiang, in the inland region of Northwest China. Based on long-term experimental data, the study explored the nutrient allometry allocation patterns among cotton organs, assessed whether composite cropping impacts nutrient limitation in cotton using allometry allocation indices and eco-chemometrics, and proposed the most suitable cultivation patterns for this region. We hypothesized the following: (1) different nutrient partitioning strategies existed among the patterns with trees, shrubs, and crops as composite plantings; (2) composite planting plants dominated by trees were more favorable to the nutrient-use efficiency of cotton; (3) available soil nutrients and pH and electrical conductivity can promote cotton nutrient uptake. The results of our study provide recommendations for solving potential challenges in cotton production and outline the future direction for cotton cultivation in the Northwest Inland Cotton Region.

## 2. Materials and Methods

### 2.1. Study Area

**Figure 1:**
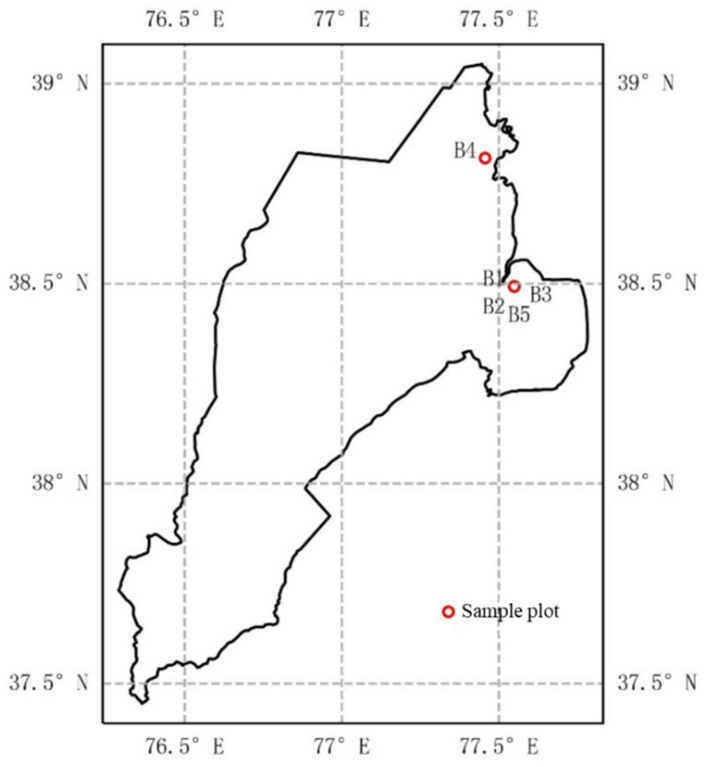
Sample site location showing the map of Shache County. B1 (cotton and three-year peach composite planting), B2 (cotton and ten-year almond tree composite planting), B3 (cotton and ten-year peach tree composite planting), B4 (cotton and three-year apple tree composite planting), and B5 (cotton and corn composite planting).

This study was conducted in Bagh Awati Township, Shache County, Xinjiang Uygur Autonomous Region (XUAR), located at 77.1° E longitude and 38.5° N latitude (Figure 1). The region has a typical temperate continental climate, with an average annual rainfall of 50 mm and an average annual temperature of 11.8 °C. Cotton is the primary cash crop in Shache County, and its cultivation heavily relies on irrigation due to the region’s scarce rainfall. According to the WRB—World Reference Base for Soil Resources 4th edition [30], the main cultivated soil type is sandy loam. The basic properties of the sandy loam soil were measured, revealing a pH of 8.42, soil organic matter content of 12.77 g/kg, and total nitrogen and available phosphorus contents of 0.73 g/kg and 9.5 mg/kg, respectively [31].

### 2.2. Experimental Setup

**Figure 2:**
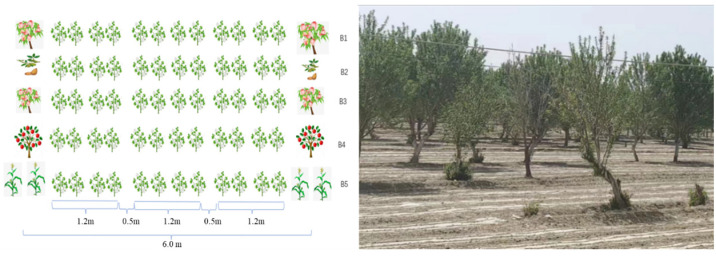
Schematic diagram of composite planting mode. B1 (cotton and three-year peach tree composite planting), B2 (cotton and ten-year almond tree composite planting), B3 (cotton and ten-year peach tree composite planting), B4 (cotton and three-year apple tree composite planting), and B5 (cotton and corn composite planting).

The intercropping experiment focused on common intercropping methods involving cotton, peach trees, and Batangas in the Shache area, based on existing local composite planting patterns with the exception of corn. Sample plots were located within the high-yield experimental demonstration plots of the Cotton Research Institute of the Chinese Academy of Agricultural Sciences (CAAS) and the Xinjiang Branch of China Cotton Group (CCMG). The cotton variety used in the experiment was 96A, provided by the Institute of Cotton Research of CAAS. Each treatment plot had a minimum area of 0.667 hm^2^, with the maximum distance between plots not exceeding 50 km (Figure 2), ensuring that the geographic location did not influence climatic differences. The surrounding terrain was open and flat, with irrigated silt soils. Detailed information on the composite cropping pattern is presented in Table 1.

The sowing, fertilization, and field management of cotton were conducted following local conventional cultivation methods.

From 20 October to 1 November 2020, straw returning, residual film cutting, and cleaning were performed. Winter irrigation took place from 25 December 2020 to 5 January 2021, with a water depth of 20 cm above the ground, resulting in an actual irrigation amount of approximately 3500 m^3^/ha. Spring irrigation occurred between 10 March and 15 2021, with an irrigation amount of 2500 m^3^/ha. From 15 April to 20 April 2021, plowing and fertilization were carried out, applying 600 kg/ha of high-quality organic fertilizer (Stanley, N + P_2_O_5_ + K_2_O ≥ 5%, organic matter content ≥ 45%), 150 kg/ha of urea (nitrogen content ≥ 46%), 300 kg/ha of calcium superphosphate (phosphorus content ≥ 46%), 150 kg/ha of potassium sulfate (potassium content ≥ 51%), and seed sowing and mulching simultaneously. A film with a thickness ≥0.01 mm and width of 120 cm was chosen, with a spacing of 50 cm between films. In the membrane four-row mode, the row spacing configuration was (20 + 50 + 20 cm), with an average row spacing of 30 cm, a plant spacing of 10 cm, and a sowing depth of 2–3 cm. Drip irrigation belts were placed in a manner of one membrane, three belts, with the belts positioned in the middle of the narrow rows.

In early May, replanting and thinning were performed based on sowing conditions. The rate of empty holes was ≤3%, and the rate of misalignment was ≤1%. The mulching thickness on the membrane was 1–2 cm, with a sowing rate of 25.5 kg/ha. From May 22 to 24, medium plowing took place, and topping occurred between 5 July and 18 when cotton reached 7–8 fruiting branching platforms. Plant height was controlled at approximately 65 cm.

From 10 May to 25 August, pesticide spraying for pest control occurred on 10 May, 20 May, 5 June, 21 June, 10 July, 5 August, and 20 August. Irrigation took place on 5 June, 20 June, 5 July, 15 July, 25 July, 5 August, 15 August, and 25 August, each lasting 8 h. Fertilizers were applied according to the soil fertility levels, with a cumulative fertilizer application of 450 kg/ha of urea, 90 kg/ha of urea phosphate, 60 kg/ha of potassium xanthate, 345 kg/ha of monoammonium phosphate, 210 kg/ of potassium sulfate, and different levels of indoxacarb sprayed before irrigation and after topping to control growth stages.

Samples were collected from 25 September to 30 September 2021, prior to defoliant spraying. Plots with uniform growth were selected and delineated as 5 m × 5 m sampling plots. Soil samples were taken by randomly selecting 2 cm of soil near the root system of multiple cotton plants and mixing them into a composite sample. The soil was then divided into two layers: 0–20 cm and 20–40 cm. Cotton plant samples were also mixed from multiple plants, with the plants divided into five parts: roots, stem (including leaves), shell, batt, and seeds. Three plots were selected for each treatment, and one sample was measured after mixing the samples from each plot.

### 2.3. Sample Determination

Soil and plant samples were sent back to the State Key Laboratory of Agriculture and Dry Areas for determination and analysis after collection. Soil carbon and plant carbon were determined using the external heating method of potassium dichromate, soil nitrogen, and plant nitrogen were determined using the Kjeldahl titration method, and soil phosphorus and plant phosphorus were determined using molybdenum antimony resisted colorimetric method with a visible light of 700 nm. Soil available phosphorus was determined using the molybdenum-antimony-resisted colorimetric method of NaHCO_3_ extraction. Soil nitrate nitrogen and ammonia nitrogen were determined using a flow analyzer [32].

### 2.4. Data Analysis

Data were organized using Excel 2016. Correlation analysis was performed using Origin 2022, histograms were plotted, and multiple comparisons were conducted using SPSS 20.0.

C-N-P partitioning relationships in different organs were analyzed using the following equation:

(1) $$Y=bX^{a},$$

where *X* and *Y* represent the C, N, and P content in the organs, respectively, and were logarithmically transformed [33].

The transformed equation was expressed as follows:

(2) $$\log\left(Y\right)=\log\left(b\right)+alog(X),$$

where *b* is the intercept, and *a* is the slope, representing the allometry allocation index. The spindle SMA estimation was performed using SMART V2.0 software, with the specific software setup interface shown in the Appendix A [34].

A significance test with slope 1 was conducted using R’s smart package.

## 3. Results

### 3.1. Effect of Composite Cropping Pattern on Soil Physicochemical Properties

Based on the results from studies conducted in the northwest inland cotton region of China between 2021 and 2022, soil conductivity in the B5 and B4 treatment was higher than in the other treatments, and the difference is more significant in the lower soil. The nitrate nitrogen content in the B5 treatment was significantly higher than other treatments, and ammonium nitrogen reached the maximum value. The available phosphorus content in the upper soil layer under the B4 treatment reached the maximum value and was significantly higher than other treatments, but there was no significant change in the lower soil layer. The pH reached the minimum value in B4, but there was no significant difference with other treatments. Soil organic carbon and total nitrogen content reached the maximum value in B2 treatment and was significantly higher than that in B3 and B4. But there was no significant difference in total phosphorus content among different treatments (Table 2). In addition, there was no consistent pattern in nutrient differences between soil layers, possibly due to the current plowing depth, which is close to or exceeds 40 cm.

### 3.2. Effects of Compound Planting Pattern on Nutrient Allocation to Major Organs of Cotton

**Figure 3:**
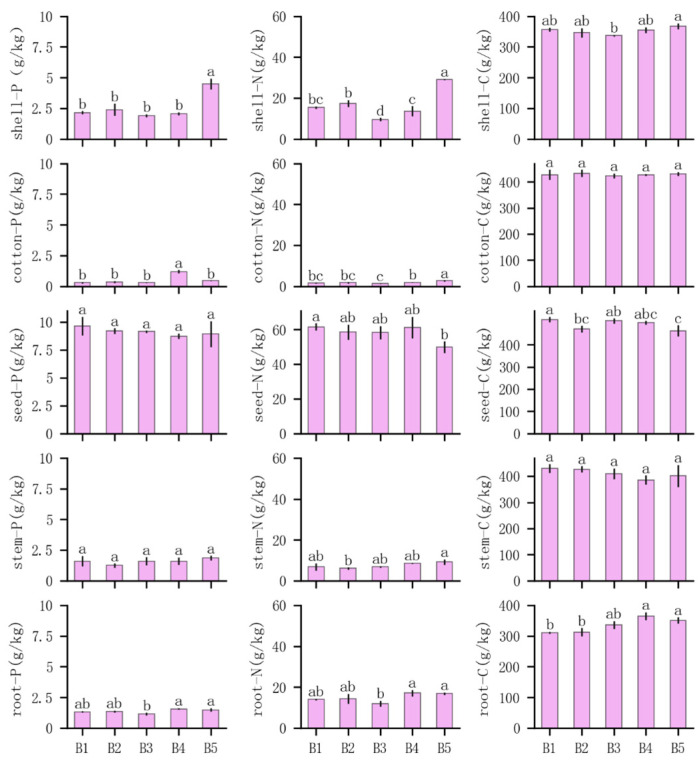
Nutrient distribution in cotton organs. Shell represents the cotton shell, cotton represents the cotton batt, seed represents the cotton seed, stem represents the cotton stem, and root represents the cotton root. C, total carbon; N, total nitrogen; P, total phosphorus. Different lowercase letters indicate significant differences between treatments (*p* < 0.05).

The phosphorus content of various cotton organs under different composite planting modes is shown in Figure 3. The phosphorus content in B5 shell was significantly higher than in the other treatments, and the phosphorus content in B4 cotton was significantly higher than in the other treatments. No significant differences were observed in phosphorus content in seed and stem, but the phosphorus content in the root system of B4 and B5 was significantly higher than in B3.

Cotton nitrogen allocation showed considerable variation. The nitrogen content in B5 shell was significantly higher than in other treatments, while B3 had significantly lower nitrogen content in cotton shell than the other treatments. The nitrogen content in B5 cotton reached the maximum value, while the nitrogen content in B3 cotton was lower than in B4 and B5. The nitrogen content in B5 stem was significantly higher than in B2, and the nitrogen content in the root system of B4 and B5 was significantly higher than in B3, while no significant differences were observed among the other treatments.

The cropping practices affected the carbon content in cotton organs. The carbon content of the stem and cotton did not differ significantly among the treatments. However, the carbon content of the root system in B4 and B5 treatments was significantly higher than in B1 and B2.

### 3.3. Composite Planting and Ecological Stoichiometry of Cotton Major Organs

**Figure 4:**
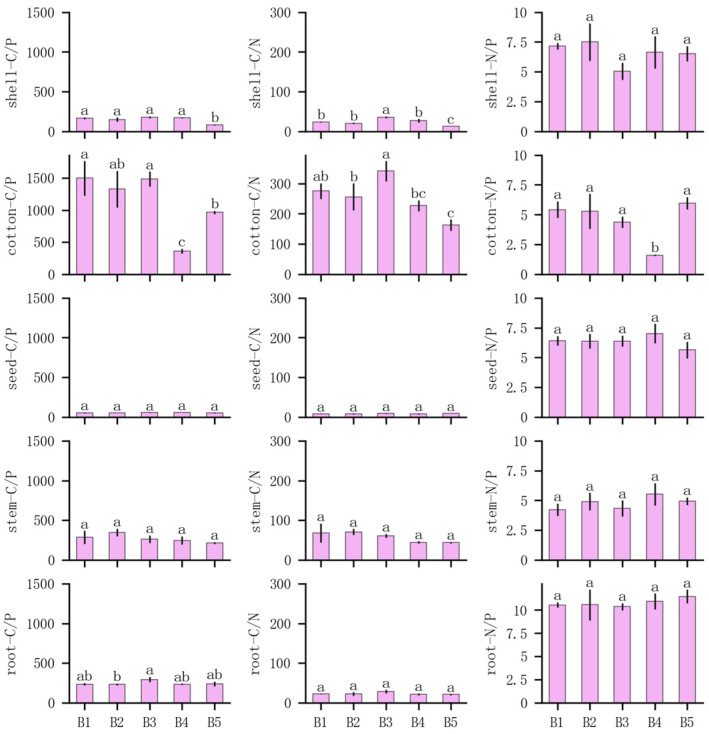
Stoichiometry of cotton organs. Shell represents the cotton shell, cotton represents the cotton batt, seed represents the cotton seed, stem represents the cotton stem, and root represents the cotton root. C/N, total carbon–total nitrogen; N/P, total nitrogen–total phosphorus; C/P, total carbon–total phosphorus. Different lowercase letters indicate significant differences between treatments (*p* < 0.05).

The ecological stoichiometry of cotton’s major organs is presented in Figure 4. The carbon-to-phosphorus ratio and carbon-to-nitrogen ratio in the cotton shells of B5 were significantly lower than those in the other treatments. Additionally, the carbon-to-phosphorus ratio and nitrogen-to-phosphorus ratio in the cotton batt of B4 were significantly lower than those of other treatments. Except for significant differences in the carbon-to-nitrogen ratio of the shell and cotton system among different composite planting treatments, the stoichiometry of the cotton stem, roots, and cotton seeds showed no significant differences.

Significant stoichiometric differences were observed among cotton organs. The carbon-to-nitrogen and carbon-to-phosphorus ratios of cotton batt were significantly higher than those of the other treatments, which were consistent with the findings of the other treatments discussed earlier.

### 3.4. Compound Planting Pattern and Nitrogen–Phosphorus Isokinetic Allocation

The effects of composite planting patterns on nitrogen and phosphorus heterozygous allocation are shown in Table 3. The *p*-values for the five composite planting patterns were all less than 0.01, reaching a highly significant level, indicating that the five treatments conformed to the heterozygous allocation model.

The slopes of B1, B2, B3, and B4 ranged from 0.97 to 0.99, and the intercepts were between 0.75 and 0.83, with minimal differences between slopes and intercepts. This suggests that the nitrogen and phosphorus heterozygous allocation levels of these four treatments were quite similar. However, significance analysis of the allometry distribution index and slope 1 showed no significant difference, indicating that the distribution of B1, B2, B3, and B4 was allometry.

The slope of B5 was 1.14, the only slope greater than 1 among the five treatments, with an intercept of −1.01, indicating differences in allocation strategies between this treatment and the others.

### 3.5. Soil–Plant Nutrient Relationship

**Figure 5:**
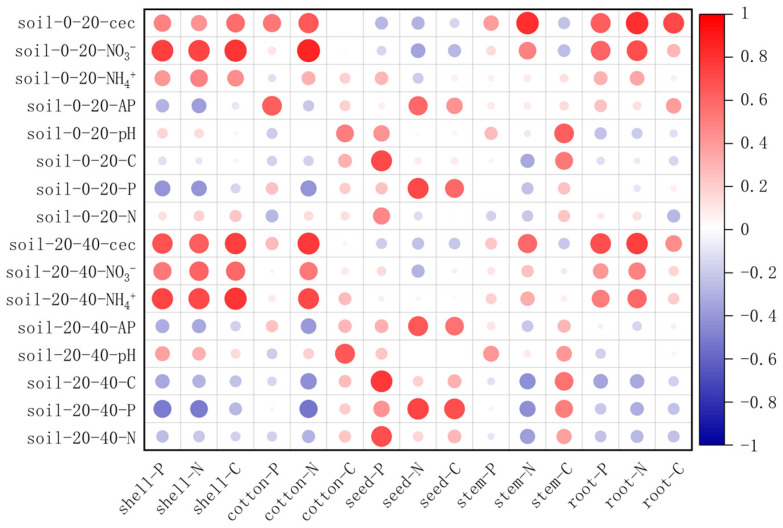
Correlation analysis between soil property and nutrient content of various organs. C, total carbon; N, total nitrogen; P, total phosphorus; NO_3_^−^, nitrate nitrogen; NH_4_^+^, ammonium nitrogen; AP, available phosphorus; pH, soil acidity and alkalinity; cec, electrical conductivity. Shell represents the cotton shell, cotton represents the cotton batt, seed represents the cotton seed, stem represents the cotton stem, and root represents the cotton root. Red circle indicates a positive correlation, while blue circle indicates a negative correlation. Different size circle represents different correlation strength, the larger the size, the stronger the correlation.

Correlation analysis results (Figure 5) showed that soil surface/subsurface conductivity and available nitrogen content were significantly positively correlated with cotton shell nutrients, among which nitrate nitrogen in the surface soil, ammonium nitrogen in the subsurface soil and electrical conductivity were significantly positively correlated with cotton root nutrients and nitrogen content of cotton stem. Soil available phosphorus and total phosphorus were significantly positively correlated with carbon and phosphorus content of cotton seed but negatively correlated with nitrogen and phosphorus content of cotton shell and nitrogen content of cotton batt. Soil organic carbon and total nitrogen were positively correlated with phosphorus content of cotton seed. Soil pH was significantly positively correlated with carbon of cotton batt and stem. Both surface and subsurface soils showed high consistency in nutrient relationships.

## 4. Discussion

Composite cropping patterns have been widely promoted in the inland cotton region of Northwest China, yet there is ongoing debate regarding nutrient competition between crops in these systems [35,36]. This study investigated nutrient partitioning and ecological stoichiometry in cotton intercropped with different plant species and found that no significant difference in cotton nutrient partitioning under tree-dominated intercropping (set-over treatment), but the phosphorus content in cotton batt was higher only in the presence of young apple trees. However, a significant difference was observed in nutrient partitioning when cotton was intercropped with corn, a graminaceous herbaceous plant. Comparison of nitrogen and phosphorus allometry partitioning models revealed that intercropping with corn resulted in distinct nitrogen and phosphorus allometry partitioning indices compared to other treatments. Ecological stoichiometry analysis indicated some effects of treatments on the carbon-to-nitrogen and carbon-to-phosphorus (C:P) ratios of cotton batt and shells. Crops or organs with higher C:N or C:P ratios tend to have higher nutrient-use efficiencies, as they can fix more carbon with fewer nutrients [37,38]. Accordingly, both cotton shell and batt in the cotton and corn composite planting exhibited lower nitrogen utilization efficiencies compared to tree-dominated intercropping. The phosphorus utilization efficiency of cotton shell in cotton and corn composite planting was significantly lower than in tree-dominated intercropping, and cotton batt in cotton and three-year apple tree composite planting showed the lowest phosphorus utilization efficiencies. The three-year apple trees were in the seedling stage, and their height and canopy size are close to that of the corn [39]. Moreover, low shrubs and herbs were less effective than tall trees for water retention and wind protection [40,41]. No significant differences in stoichiometry were observed in the roots and stems, suggesting that composite cropping patterns primarily influence nutrient-use efficiency during the reproductive growth stage of organ differentiation [42,43].

Correlation analysis revealed that soil conductivity and available nitrogen content were positively correlated with nutrient partitioning in the roots, cotton shell, and cotton itself, with no significant difference between surface and subsurface soils [44]. Conductivity generally reflects soil salinity, and high salt content is typically detrimental to plant growth [45,46]. Thus, the positive correlation between conductivity and root nutrient content may represent a protective response to high salinity environments. Available-acting phosphorus was positively correlated with phosphorus content in all cotton organs, whereas total phosphorus content did not show a significant relationship with phosphorus partitioning in the organs. This suggests that available-acting phosphorus, which is more readily available to plants, provides a better indication of phosphorus absorption and utilization [47,48,49]. pH had a notable effect on cotton batt and stem nutrient content. In the alkaline soil of the northwest inland cotton region, high pH levels directed more nutrients to the stems during the vegetative growth stage and to the seeds during the reproductive growth stage, thus enhancing seed viability.

## 5. Conclusions

This study employed a controlled experiment to examine cotton nutrient partitioning under various composite cropping patterns. The results revealed that the nutrient distribution of cotton was of equal speed under the combined planting with trees, while there was an allometric distribution index of N and P between the combined planting with maize. The effect of compound planting mode on nutrient-use efficiency of cotton was mainly reflected in the organ differentiation stage of its reproductive growth stage. Cotton showed lower nutrient-use efficiency in reproductive organs when intercropped with low shrubs and herbaceous crops, likely due to the lower protective capacity of these plants. Intercropping with tall trees, however, had a positive effect on cotton’s nutrient utilization efficiency. Despite this, tall tree intercropping also reduced the nitrogen and phosphorus content in cotton batt. Soil available nitrogen and conductivity positively influenced the nutrient uptake of cotton shell and roots, while soil phosphorus promoted the nutrient absorption of cotton seed but inhibited the nitrogen and phosphorus of cotton shell and the nitrogen of cotton batt.

## Figures and Tables

**Table 1:** Detailed information of composite planting modes.

Group	Planting Patterns	Planting Time
B1	Peach trees are planted with a row spacing of 6.0 m and a plant spacing of 4.0 m, with cotton planted in between rows.	2018
B2	Almond trees are planted with a row spacing of 6.1 m and a plant spacing of 4.8 m, with cotton planted in between rows.	2011
B3	Peach trees are planted with a row spacing of 6.0 m and a plant spacing of 4.5 m, with cotton planted in between rows.	2011
B4	Apple trees are planted with a row spacing of 6.0 m and a plant spacing of 4.2 m, with cotton planted in between rows.	2018
B5	At intervals of 5.4 m, two rows of corn are planted with a row spacing of 60 cm and a plant spacing of 20 cm	2021

**Table 2:** Main physical and chemical property indexes of soil.

Indexes	Soil Layer (cm)	Compound Planting Mode				
		B1	B2	B3	B4	B5
EC (μs/cm)	0–20	519.33 ± 62.37 b	698.33 ± 38.78 b	928.33 ± 100.25 b	2078.67 ± 265.26 a	2415.67 ± 370.62 a
	20–40	591.67 ± 87.79 d	1099.67 ± 251.1 c	927.33 ± 120.33 c	1720.33 ± 263.22 b	2682.00 ± 121.96 a
NO_3_^−^-N (mg/kg)	0–20	11.50 ± 1.60 c	34.22 ± 6.79 b	5.14 ± 1.10 c	57.85 ± 10.54 b	144.33 ± 25.79 a
	20–40	29.31 ± 2.04 c	53.91 ± 8.26 b	4.77 ± 0.36 d	21.72 ± 9.12 c	105.81 ± 19.46 a
NH_4_^+^-N (mg/kg)	0–20	1.06 ± 0.45 a	0.91 ± 0.69 a	0.39 ± 0.01 b	0.56 ± 0.01 b	1.38 ± 0.55 a
	20–40	0.57 ± 0.28 b	1.12 ± 0.01 a	0.50 ± 0.02 b	1.00 ± 0.02 a	1.61 ± 0.58 a
AP (mg/kg)	0–20	11.10 ± 2.07 bc	14.43 ± 0.71 b	9.37 ± 0.61 bc	22.04 ± 2.23 a	8.11 ± 1.29 c
	20–40	10.88 ± 2.70 a	13.58 ± 1.37 a	13.48 ± 0.88 a	13.34 ± 3.52 a	9.40 ± 1.49 a
pH	0–20	8.34 ± 0.05 a	8.25 ± 0.09 a	8.23 ± 0.22 a	7.85 ± 0.03 a	8.34 ± 0.18 a
	20–40	8.22 ± 0.26 a	8.22 ± 0.15 a	8.16 ± 0.14 a	7.98 ± 0.17 a	8.33 ± 0.16 a
SOC (g/kg)	0–20	6.52 ± 0.61 ab	9.24 ± 1.31 a	4.92 ± 0.54 b	4.30 ± 0.12 b	6.02 ± 0.56 ab
	20–40	7.62 ± 0.98 a	7.20 ± 0.58 a	6.01 ± 0.34 ab	4.41 ± 0.46 b	5.53 ± 0.89 ab
TP (g/kg)	0–20	0.91 ± 0.07 a	0.87 ± 0.10 a	0.82 ± 0.06 a	0.95 ± 0.05 a	0.75 ± 0.05 a
	20–40	1.03 ± 0.16 a	0.93 ± 0.16 a	0.85 ± 0.02 ab	0.85 ± 0.03 ab	0.70 ± 0.07 b
TN (g/kg)	0–20	0.73 ± 0.07 b	0.95 ± 0.0 9a	0.44 ± 0.01 b	0.58 ± 0.03 b	0.74 ± 0.11 ab
	20–40	0.88 ± 0.04 a	0.82 ± 0.10 a	0.59 ± 0.09 b	0.55 ± 0.04 b	0.67 ± 0.17 ab

Note: SOC, soil organic carbon; TN, total nitrogen; NO_3_^−^-N, nitrate nitrogen; NH_4_^+^-N, ammonium nitrogen; TP, total phosphorus; AP, available phosphorus; pH, soil acidity and alkalinity; EC, electrical conductivity. Different lowercase letters indicate significant differences between different compound planting modes (*p* < 0.05).

**Table 3:** Intercropping methods and allometric distribution of nitrogen and phosphorus.

Group	*n*	R2	Slope	LowCI	UppCI	Interc	LowCI	UppCI	Ymean	Xmean	*p*
B1	15	0.837	0.9708	0.7333	1.2853	−0.7907	−1.1445	−0.4369	0.408	1.234	**
B2	15	0.862	0.9907	0.7646	1.2838	−0.8332	−1.1659	−0.5005	0.39	1.235	**
B3	15	0.83	0.9726	0.7305	1.295	−0.7567	−1.0994	−0.414	0.374	1.162	**
B4	15	0.853	0.98	0.7503	1.2799	−0.8331	−1.1794	−0.4868	0.412	1.271	**
B5	15	0.791	1.1385	0.8293	1.5631	−1.0136	−1.5143	−0.5129	0.509	1.338	**

Note: *n* represents sample size, R^2^ represents coefficient of determination, Slope represents degree of skewness, LowCI represents lower limit of confidence interval for slope, UppCI represents upper limit of confidence interval for slope, Interc represents intercept, The second LowCI represents lower limit of confidence interval of the intercept, The second UppCI represents upper limit of confidence interval of the intercept, Ymean represents mean of *Y* represents, Xmean represents mean of *X*, and *p* represents significance (** *p*<0.01 is extremely significant).

## Data Availability

The original contributions presented in this study are included in the article. Further inquiries can be directed to the corresponding author. The data are not publicly available due to privacy issues.

## Appendix A

### SMRAT Software Setup Interface

where I is the data source file name and O is the data output file name.

where G, Y, X are the data headers, and T selects log Y vs. log X.

## References

[1] Berg G. (2009). Plant–microbe interactions promoting plant growth and health: Perspectives for controlled use of microorganisms in agriculture. Appl. Microbiol. Biotechnol..

[2] Pretty J., Bharucha Z.P. (2014). Sustainable intensification in agricultural systems. Ann. Bot..

[3] Ratnadass A., Fernandes P., Avelino J., Habib R. (2012). Plant species diversity for sustainable management of crop pests and diseases in agroecosystems: A review. Agron. Sustain. Dev..

[4] Li C., Stomph T.J., Makowski D., Li H., Zhang C., Zhang F., Van der Werf W. (2023). The productive performance of intercropping. Proc. Natl. Acad. Sci. USA.

[5] Wu Y., Wang E., Gong W., Xu L., Zhao Z., He D., Yang F., Wang X., Yong T., Liu J. (2023). Soybean yield variations and the potential of intercropping to increase production in China. Field Crops Res..

[6] Chen G., Jiang F., Zhang S., Zhang Q., Jiang G., Gao B., Cao G., Islam M.U.I., Cao Z., Zhao X. (2025). Potential crop yield gains under intensive soybean/maize intercropping in China. Plant Soil.

[7] Kebede E. (2021). Contribution, Utilization, and Improvement of Legumes-Driven Biological Nitrogen Fixation in Agricultural Systems. Front. Sustain. Food Syst..

[8] Yang T., Ouyang X., Wang B., Tian D., Xu C., Lin Z., Ge X., Tang L. (2023). Understanding the effects of tree-crop intercropping systems on crop production in China by combining field experiments with a meta-analysis. Agric. Syst..

[9] Feng L., Dai J., Tian L., Zhang H., Li W., Dong H. (2017). Review of the technology for high-yielding and efficient cotton cultivation in the northwest inland cotton-growing region of China. Field Crops Res..

[10] Li N., Li Y., Biswas A., Wang J., Dong H., Chen J., Liu C., Fan X. (2021). Impact of climate change and crop management on cotton phenology based on statistical analysis in the main-cotton-planting areas of China. J. Clean. Prod..

[11] Govereh J., Jayne T.S. (2003). Cash cropping and food crop productivity: Synergies or trade-offs?. Agric. Econ..

[12] Grassini P., Cassman K.G. (2012). High-yield maize with large net energy yield and small global warming intensity. Proc. Natl. Acad. Sci. USA.

[13] Zhang W., Wang B.J., Gan Y.W., Duan Z.P., Hao X.D., Xu W.L., Li L.H. (2019). Competitive interaction in jujube tree/cotton agroforestry system in Xinjiang province, northwestern China. Agrofor. Syst..

[14] Bai T., Zhang N., Wang T., Wang D., Yu C., Meng W., Fei H., Chen R., Li Y., Zhou B. (2021). Simulating on the effects of irrigation on jujube tree growth, evapotranspiration and water use based on crop growth model. Agric. Water Manag..

[15] Ai P., Ma Y., Hai Y. (2021). Influence of jujube/cotton intercropping on soil temperature and crop evapotranspiration in an arid area. Agric. Water Manag..

[16] Zhang X., Zhang J., Khan A., Zhu D., Zhang Z. (2024). Improving the productivity of Xinjiang cotton in heat-limited regions under two life history strategies. J. Environ. Manag..

[17] Shah M.A., Farooq M., Hussain M. (2016). Productivity and profitability of cotton–wheat system as influenced by relay intercropping of insect resistant transgenic cotton in bed planted wheat. Eur. J. Agron..

[18] Matloob A., Aslam F., Rehman H.U., Khaliq A., Ahmad S., Yasmeen A., Hussain N., Ahmad S., Hasanuzzaman M. (2020). Cotton-Based Cropping Systems and Their Impacts on Production. Cotton Production and Uses: Agronomy, Crop Protection, and Postharvest Technologies.

[19] Peixi S., Tingting X. (2016). Agroecology Research and Practice in the Oasis Region, Northwest China. Agroecology in China.

[20] Tian P. (2021). A New Theory Producing Heterosis of Crops. Open Access Libr. J..

[21] Guo J., Jia Y., Chen H., Zhang L., Yang J., Zhang J., Hu X., Ye X., Li Y., Zhou Y. (2019). Growth, photosynthesis, and nutrient uptake in wheat are affected by differences in nitrogen levels and forms and potassium supply. Sci. Rep..

[22] Veresoglou S.D., Peñuelas J., Fischer R., Rautio P., Sardans J., Merilä P., Tabakovic-Tosic M., Rillig M.C. (2014). Exploring continental-scale stand health—N:P ratio relationships for European forests. New Phytol..

[23] Ekvall L., Greger M. (2003). Effects of environmental biomass-producing factors on Cd uptake in two Swedish ecotypes of Pinus sylvestris. Environ. Pollut..

[24] Veresoglou S.D., Peñuelas J. (2019). Variance in biomass-allocation fractions is explained by distribution in European trees. New Phytol..

[25] Tang X., Xia M., Guan F., Fan S. (2016). Spatial Distribution of Soil Nitrogen, Phosphorus and Potassium Stocks in Moso Bamboo Forests in Subtropical China. Forests.

[26] Cai X., Lin Z., Penttinen P., Li Y., Li Y., Luo Y., Yue T., Jiang P., Fu W. (2018). Effects of conversion from a natural evergreen broadleaf forest to a Moso bamboo plantation on the soil nutrient pools, microbial biomass and enzyme activities in a subtropical area. For. Ecol. Manag..

[27] Liu Y., Zhao X., Liu W., Feng B., Lv W., Zhang Z., Yang X., Dong Q. (2024). Plant biomass partitioning in alpine meadows under different herbivores as influenced by soil bulk density and available nutrients. CATENA.

[28] Wang Q., Wang J., Huang X., Liu Z., Jin W., Hu W., Meng Y., Zhou Z. (2024). Phosphorus application under continuous wheat-cotton straw retention enhanced cotton root productivity and seedcotton yield by improving the carbohydrate metabolism of root. Field Crops Res..

[29] Hou X., Li H., Ding R., Du T. (2024). Hydraulic, morphological, and anatomical changes over the development of cotton bolls and pedicels leading to boll opening under well-watered and water deficit conditions. Environ. Exp. Bot..

[30] Schad P. (2023). World Reference Base for Soil Resources—Its fourth edition and its history. J. Plant Nutr. Soil Sci..

[31] Rao X., Wang Z., Geng Q., Li X., Chen S. (2014). The influence of topography on the quality of cultivated land and its improvement measures in Shache County. J. Agric. Sci..

[32] Bao S. (2000). Soil Agrochemical Analysis.

[33] Gao X., Koven C.D., Kueppers L.M. (2024). Allometric relationships and trade-offs in 11 common mediterranean-climate grasses. Ecol. Appl..

[34] Hafner J.M., Steinke J., Uckert G., Sieber S., Kimaro A.A. (2021). Allometric equations for estimating on-farm fuel production of gliricidia sepium (gliricidia) shrubs and cajanus cajan (pigeon pea) plants in semi-arid tanzania. Energy Sustain. Soc..

[35] Huang W., Wu F., Han W., Li Q., Han Y., Wang G., Feng L., Li X., Yang B., Lei Y. (2022). Carbon footprint of cotton production in China: Composition, spatiotemporal changes and driving factors. Sci. Total Environ..

[36] Zhou Y., Li F., Xin Q., Li Y., Lin Z. (2024). Historical variability of cotton yield and response to climate and agronomic management in Xinjiang, China. Sci. Total Environ..

[37] Zhang Q., Li G., Lu W., Lu D. (2022). Interactive Effects of Nitrogen and Potassium on Grain Yield and Quality of Waxy Maize. Plants.

[38] Liu X., Meng L., Yin T., Wang X., Zhang S., Cheng Z., Ogundeji A.O., Li S. (2023). Maize/soybean intercrop over time has higher yield stability relative to matched monoculture under different nitrogen-application rates. Field Crops Res..

[39] Zhou X., Wang R., Gao F., Xiao H., Xu H., Wang D. (2019). Apple and maize physiological characteristics and water-use efficiency in an alley cropping system under water and fertilizer coupling in Loess Plateau, China. Agric. Water Manag..

[40] Wei W., Wang B., Niu X. (2020). Soil Erosion Reduction by Grain for Green Project in Desertification Areas of Northern China. Forests.

[41] Zhu J., Song L. (2021). A review of ecological mechanisms for management practices of protective forests. J. For. Res..

[42] Song Y., Yu Y., Li Y. (2022). Leaf Functional Traits and Relationships with Soil Properties of Zanthoxylum planispinum ‘dintanensis’ in Plantations of Different Ages. Agronomy.

[43] Fu X., Ma Y., Yang T., He S., Wang D., Jin L., Zhan L., Guo Z., Fan K., Li J. (2024). Bacterial community composition of wheat aboveground compartments correlates with yield during the reproductive phase. Appl. Environ. Microbiol..

[44] Akça E., Aydin M., Kapur S., Kume T., Nagano T., Watanabe T., Çilek A., Zorlu K. (2020). Long-term monitoring of soil salinity in a semi-arid environment of Turkey. CATENA.

[45] Monteiro L.C.P., Matos C.d.C.d., Cândido A.d.O., Mendes T.A.d.O., Santana M.F., Costa M.D. (2021). Changes in rhizosphere microbial diversity and composition due to NaCl addition to the soil modify the outcome of maize-weed interactions. Appl. Soil Ecol..

[46] Iseas M.S., Sainato C.M., Gómez A., Romay C. (2024). Assessing salinity and sodicity of irrigated soils using apparent electrical conductivity in the Pampean region. Environ. Earth Sci..

[47] Kleinman P.J.A., Sharpley A.N., Wolf A.M., Beegle D.B., Moore P.A. (2002). Measuring Water-Extractable Phosphorus in Manure as an Indicator of Phosphorus in Runoff. Soil Sci. Soc. Am. J..

[48] Oliveira V., Horta C., Dias-Ferreira C. (2019). Evaluation of a phosphorus fertiliser produced from anaerobically digested organic fraction of municipal solid waste. J. Clean. Prod..

[49] Wissuwa M., Gonzalez D., Watts-Williams S.J. (2020). The contribution of plant traits and soil microbes to phosphorus uptake from low-phosphorus soil in upland rice varieties. Plant Soil.

